# Disentangling effects of the DR and DQ isomers encoded by the HLA class II haplotype DRB1*15:01/DQB1*06:02 to help establish the true risk allele for FVIII inhibitor development in Hemophilia A

**DOI:** 10.3389/fgene.2025.1506862

**Published:** 2025-04-09

**Authors:** Vincent P. Diego, Bernadette W. Luu, Marcio A. Almeida, Raja Rajalingam, Marco Hofmann, Jacob A. Galan, Eron G. Manusov, Jerry S. Powell, Long V. Dinh, Henry Mead, Huy Huynh, Anne M. Verhagen, Juan M. Peralta, Paul V. Lehmann, Satish Kumar, Eli J. Fine, Joanne E. Curran, Harald H. Goring, Miguel A. Escobar, Sarah Williams-Blangero, Eugene Maraskovsky, John Blangero, Tom E. Howard

**Affiliations:** ^1^ South Texas Diabetes and Obesity Institute, and Division of Human Genetics, Department of Primary and Community Care, School of Medicine, University of Texas Rio Grande Valley, Brownsville, TX, United States; ^2^ Haplogenics Corporation, Brownsville, TX, United States; ^3^ Immunogenetics and Transplantation Laboratory, Department of Surgery, School of Medicine, University of California at San Francisco, San Francisco, CA, United States; ^4^ CSL Innovation GmbH, Marburg, Germany; ^5^ Division of Hematology and Oncology, Department of Internal Medicine, School of Medicine, University of California at Davis, Davis, CA, United States; ^6^ Global Medical Affairs, BioMarin, Novato, CA, United States; ^7^ CSL Limited Research, Bio21 Institute, Melbourne, VIC, Australia; ^8^ Cellular Technology Ltd, Shaker Heights, OH, United States; ^9^ Departments of Pathology and Neurology, Case Western Reserve University School of Medicine, Cleveland, OH, United States; ^10^ Fine Consultancy, Smyrna, GA, United States; ^11^ Division of Hematology and Oncology, Department of Medicine, University of Texas Health Science Center, and Gulf States Hemophilia and Thrombophilia Center, Houston, TX, United States; ^12^ Department of Pathology and Laboratory Medicine, VA-Valley Coastal Bend Healthcare System, Harlingen, TX, United States

**Keywords:** Hemophilia A, therapeutic FVIII proteins, FVIII inhibitors, linkage disequilibrium and HLAII haplotype DRB1*15:01/DQB1*06:02, dendritic cell protein processing and presentation assays, therapeutic FVIII derived peptides, MHC-associated peptide proteomics, immunogenic potential

## Abstract

**Introduction:**

Hemophilia A (HA) patients (HAPs) with the human leukocyte antigen (HLA)-class-II (HLAII) haplotype DRB1*15:01/DQB1*06:02, and thus antigen presenting cells which express HLAII β-polypeptide chains that form heterodimers of DR15- and DQ6-serotypes, respectively, have an increased risk of developing factor (F)VIII inhibitors (FEIs)—neutralizing antibodies against the therapeutic-FVIII-proteins (tFVIIIs) infused to prevent/arrest bleeding. As DRB1*15:01 and DQB1*06:02 exist in strong linkage disequilibrium, association analysis cannot determine which is the actual risk allele.

**Methods:**

To establish the true risk allele of this haplotype, we analyzed the tFVIII-derived peptides (tFVIII-dPs) bound to either the DR or DQ molecules that comprise the individual HLAII repertoires expressed by monocyte-derived dendritic cells obtained from 25 normal blood donors and six HAPs, four without and two with FEIs. We performed log-linear mixed model analyses, where the dependent variable is the log of the measured peptide count. Under Model 1, we analyzed an HLAII allele predictor consisting of ten levels (four DRB1 and six DQB1 alleles) in the fixed effects and variables in the random effects to account for non-independence. Model 2—where the HLAII allele variable consisted of only DRB1*15:01 and DQB1*06:02—compares the HLAII alleles.

**Results:**

Relative to the Model 1 reference, DRB1*15:01 and DQB1*06:02 significantly increased tFVIII-derived peptide counts, and DRB1*15:01 contributed significantly more than DQB1*06:02. Reported as risk ratios (RRs) and their 95% confidence interval (CI) lower- (LB) and upper-bound (UB), we found a RR (95% CI-LB, -UB) of 14.16 (10.38, 19.33) and 1.76 (1.24, 2.50) for DRB1*15:01 and DQB1*06:02, respectively. Under Model 2, we found an RR for DRB1*15:01 against DQB1*06:02 of 7.00 (5.80, 8.44).

**Discussion/conclusion:**

Our results suggest that DRB1*15:01 is the offending HLAII allele and that DR15 allotypes underlie the increased FEI risk in HAPs.

## Introduction

Hemophilia A (HA) is the X-linked bleeding disorder that results from highly heterogeneous loss-of-function factor (F) VIII (FVIII) gene (*F8*) mutations and variably deficient-to-absent plasma FVIII coagulant activity (FVIII:C). Life-long regularly repeated infusions of plasma-derived (pd) or recombinant (r) therapeutic FVIII replacement proteins (tFVIIIs) are the standard of care for preventing and arresting bleeding in HA patients (HAPs), but ∼25% will develop neutralizing anti-tFVIII-antibodies, called “FVIII inhibitors (FEIs),” which impair or eliminate their efficacy (Lai and Lillicrap, 2017; Cormier et al., 2020). In addition to well-known environmental variables, FEI risk is influenced by genetic variables, which include the highly heterogeneous set of causative *F8* mutations; the functionally distinct single-nucleotide variations (SNVs) in or near loci critical for immune responses which are frequently called immune-mediated disease (IMD) genes; haplotypes of nonsynonymous (ns) SNVs in genes that encode (i) the HLA-class-II (HLAII) molecules used by dendritic cells (DCs), B-cells, and macrophages to present peptide antigens to T cells (i.e., *DPA1*, *DPB1*, *DQA1*, *DQB1*, *DRB1*, *DRB3*, *DRB4*, and *DRB5*), and (ii) all or part of FVIII (i.e., *F8*, *F8*_*I22I*_, and *F8*_*B*_) (Howard et al., 2011; Margaglione and Intrieri, 2018).

In this study, we focused on the HLAII system, particularly on the Chr6 haplotype DRB1*15:01/DQB1*06:02, which contains the *15:01 and *06:02 alleles of *DRB1* and *DQB1* that exist in strong linkage disequilibrium (LD). This haplotype was found in early studies of HAPs to be positively associated with the risk of developing FEIs (Pavlova et al., 2009). While more recent studies have provided additional support for DRB1*15:01, none have reported that DQB1*06:02 influences FEI risk (Diego et al., 2020; Lessard et al., 2022). Moreover, DRB1*15:01 has also been implicated in the risk for developing autoimmune disorders, such as multiple sclerosis and type 1 diabetes, as well as drug-induced immune-mediated liver disease (Hollenbach and Oksenberg, 2015; Hu et al., 2015; Mangalam et al., 2013; Singer et al., 2010). It cannot be definitively concluded that DRB1*15:01 is the sole risk allele based on genetic association studies alone, however, as it exists in LD with DQB1*06:02. This is especially so in light of findings from a study involving a mouse model of multiple sclerosis in which humanized mice expressing only DQB1*06:02 develop a multiple sclerosis-like neurodegenerative disease mediated by autoimmune targeting of myelin-oligodendrocyte-basic-protein (Kaushansky and Ben-Nun, 2014).

To answer this question and better understand T-cell epitope generation and presentation, we used DC-protein processing and presentation assays (PPPAs), followed by mass spectrometric and peptide proteomic analyses called “MHC-associated peptide proteomics (MAPPs)” to identify and quantify the HLAII-bound/tFVIII-derived peptides (dPs) (Diego et al., 2020; van Haren et al., 2011; V et al., 2013; Sorvillo et al., 2016; Peyron et al., 2018; Jankowski et al., 2019). The DC-PPPA data generated are the HLAII-bound/tFVIII-dPs, with the total number of peptides derived from a specific tFVIII by the DCs of a given HAP, being directly proportional to its immunogenic potential (IP) in that subject (Diego et al., 2020; Jankowski et al., 2019). Because DC-PPPAs and MAPPs analyses yield HLAII-isomer-specific peptide counts—the number of HLAII-bound/tFVIII-dPs being determined for the DP-, DQ-, and DR-isomers separately—we used a generalized linear mixed model to analyze the data (after organization into multiway contingency tables) and draw inferences regarding the relative importance of the DQ- and DR-allotypes to IP. We analyzed HLAII-bound tFVIII-derived peptidomic profiling data generated from DC-PPPAs performed previously in three independent experiments on both the HLA-DQ and -DR isomers (Peyron et al., 2018; Jankowski et al., 2019; Diego et al., 2020). The tFVIII in all three experiments was a full-length (FL)-r-tFVIII, designated “FL-rFVIII” herein.

## Methods

### Subjects and samples

As described by Peyron et al. (2018), Jankowski et al. (2019), and Diego et al. (2020), peripheral blood mononuclear cells (PBMCs) were isolated from six HAPs—including four without and two with FEIs designated FEI− and FEI+, respectively—and 25 normal donors (NDs) for use in three independent DC-PPPAs (see DC-PPPAs and MAPPs). Information concerning the required Institutional Review Board (IRB) approvals and informed consent have been detailed previously (Peyron et al., 2018; Jankowsky et al., 2019; Diego et al., 2020). Briefly, IRB approval was obtained at either: (1) the University of North Carolina Chapel Hill (USA), which is where PBMCs were isolated from component blood samples obtained from the six HAPs by cytopheresis after each provided informed consent (Jankowski et al., 2019; Diego et al., 2020); (2) Sanquin Research Laboratories (Amsterdam, NLD), where PBMCs were isolated from whole blood (WB) samples obtained by phlebotomy from the nine NDs (Peyron et al., 2018); or (3) Addenbrooke's Hospital (Cambridge, GBR), where PBMCs were isolated from WB samples obtained by phlebotomy from the 16 NDs (Jankowski et al., 2019; Diego et al., 2020). All six HAPs were Caucasian, as detailed in Jankowski et al. (2019), which additionally provides the relevant characteristics of these subjects necessary for clinical/pathologic correlation. Though the racial ancestry of the nine NDs studied by Peyron et al. (2018) and the 16 NDs studied by Jankowski et al. (2019) and Diego et al. (2020) is unknown—as the Ethics Committee at the Sanquin Research Laboratories and Addenbrooke's Hospital, respectively, did not allow this information to be given to the investigators or, for the latter, to ProImmune (www.ProImmune.com) (Oxford, GBR), the fee-for-service company which performed the DC-PPPAs reported in Jankowski et al. (2019) and Diego et al. (2020), i.e., after receiving their WB samples, isolating PBMCs then monocytes, and differentiating their monocytes into immature DCs—most were likely to be Caucasian based on the demographics of US and European blood donors, which, as reported by Wang et al. (2004), Garratty et al. (2004), Glynn et al. (2006), and Benjamin et al. (2008), are typically greater than 80% white. Important details concerning the collection, processing, shipping, and DNA extraction of/from PBMCs have also been described previously by Peyron et al. (2018), Jankowski et al. (2019), and Diego et al. (2020).

### DC-PPPAs and MAPPs

The DC-PPPAs used for the research described herein—and in prior investigations referred to collectively as the “FED (FVIII Epitope Determination) Study”—was selected because it directly identifies and relatively quantifies the HLAII-bound/tFVIII-dPs presented by DCs after their uptake and processing of tFVIIIs, doing so separately for the three isomeric forms of HLAII molecules, including DP, DQ, and DR (Diego et al., 2020; Peyron et al., 2018; Jankowski et al., 2019). Note that for the purpose of maximizing our ability to disentangle the allele-specific effects of the DRB1*15:01/DQB1*06:02 haplotype by combining our data with those of Peyron et al. (2018), we were not able to address any DP isomer effects as this other study only isolated/identified the DR- and DQ-bound tFVIII-dPs. The two independent DC-PPPAs and MAPPs analyses reported in Jankowski et al. (2019) and Diego et al. (2020) were conducted under identical experimental conditions at ProImmune using (i) their ProPresent Antigen Presentation Assay (Ventura et al., 2012; Xue et al., 2016; Gouw et al., 2018), (ii) a FL-r-tFVIII which originated as Advate® (Takeda) that we designated “FL-rFVIII”, and (iii) monocyte-derived DCs that were obtained from two different cohorts—one with 12 NDs and the other with four NDs and six HAPs—which are referred to as “S1” and “S2” in Diego et al. (2020). Briefly, immature DCs, which were differentiated from the monocytes isolated from each subject’s PBMCs, were cultured for 7 days with 146 nM of FL-rFVIII in both DC-PPPA-S1 and DC-PPPA-S2 (Diego et al., 2020; Jankowski et al., 2019). After the DCs were induced to maturity with lipopolysaccharide (LPS) overnight, harvested, washed, and then detergent-lysed, their HLAII molecules were affinity-purified as two separate isomer fractions with proprietary anti-DQ and anti-DR monoclonal antibodies whose binding properties were established by ProImmune to be equivalent to the mouse monoclonal antibodies used previously for DC-PPPAs and MAPPs analyses (van Haren et al., 2011; Van Haren et al., 2013; Sorvillo et al., 2016; Peyron et al., 2018; Ventura et al., 2012; Xue et al., 2016; Gouw et al., 2018; van Haren et al., 2012), which include anti-DQ (SPV-L3) (Novus Biologicals) and anti-DR (L243) (Abcam). Peptides eluted from the DQ or DR molecules were analyzed by high-resolution sequencing mass spectrometry (MS), designated herein as liquid chromatography (LC)-tandem MS (LC-MS/MS). The set of tFVIII-derived individual peptide sequences (IPSs) were identified by MAPPs analyses using software to compare the resulting MS data against the UniProt SWISSProt Reference Human Proteome Database supplemented with the amino acid sequences of the commercially available r-tFVIIIs and the known *F8* nonsynonymous (ns)-SNPs (Diego et al., 2020).

The DC-PPPAs and MAPPs analyses performed in Peyron et al. (2018)–which were conducted in the laboratories of Drs. Jan Voorberg and Sander Meijer at Sanquin (Amsterdam, NLD) utilizing the same FL-r-tFVIII that originated as Advate® and thus is also designated “FL-rFVIII herein”–used monocyte-derived DCs generated from nine NDs. After pulsing with 100 nM of FL-rFVIII, these DCs were then induced to maturity overnight with LPS, which stabilizes the expression of HLAII-peptide complexes on their cell surface. Analogous to that described above for Diego et al. (2020), the cells were subsequently lysed and their HLA-DR or -DQ molecules were purified using Sepharose beads conjugated with the monoclonal antibodies L243 (DR) or SPV-L3 (DQ). Samples were then analyzed using LC-MS/MS.

As detailed previously in Jankowski et al. (2019), quality control (QC) of the DC-PPPA/MAPPs experiments performed involved confirming that the immature and mature DCs expressed DC markers CD86, DC-SIGN and HLA-DR using immunocytochemistry and flow cytometry (data not shown) and identifying (in the separate DQ- and DR-peptidomes analyzed) HLAII-bound IPSs derived from endogenous proteins known to reside in the endoplasmic reticulum, Golgi, and/or endolysosomal compartment. Specifically, for each of the two isomer groups of HLAII molecules in an experiment—for example, the DR-peptidome of a subject’s DCs cultured with FL-rFVIII—to pass QC within the collection of IPSs identified, some had to have arisen from a minimum of three of the following six endogenous proteins: invariant chain, lysosome-associated membrane proteins-1/-3, transferrin receptor, FCER2/FCGR2, integrin-α_M_, and apo-lipoprotein B (Diego et al., 2020; Peyron et al., 2018; Jankowski et al., 2019). The likelihood of an LC-MS/MS-identified-peptide being a real identity is described by its expected value and the false discovery rate (Xue et al., 2016; Jeong et al., 2012). In Jankowski et al. (2019), we described how the 1) scoring algorithms and statistical significance determinations were used, 2) residues in tFVIII-derived-IPSs were numbered, and 3) FVIII-derived-IPSs were counted if they contained a minor allele(s) at a variable residue(s). To replicate the DC-PPPAs/MAPPs analyses, a request may be submitted to ProImmune to perform ProPresent assays using the same tFVIIIs and experimental conditions, and similar cellular samples from comparable NDs and HAPs. A similar QC procedure for the DC-PPPA/MAPPs experiments performed at Sanquin are detailed in Peyron et al. (2018)

### Statistical analysis

We performed log-linear mixed model analyses under two models, where the dependent variable in both models is the logarithm of the expected peptide count (Knudson et al., 2021; Woodward, 2013; Fienberg and Rinaldo, 2012). Under Model 1, we analyzed in the fixed-effect component of the model a single HLAII-allele predictor variable consisting of ten levels represented by distinct DRB1 and DQB1 alleles (four and six levels, respectively). Under this model, we analyzed in the random effects component of the model variables for individuals (eight and nine levels for Peyron et al. (2018) and Diego et al. (2020), respectively), experiments (two levels for Peyron et al. (2018) and Diego et al. (2020)), and HLAII isomers (two levels for DQ and DR) (Peyron et al., 2018; Jankowski et al., 2019; Diego et al., 2020). The random effects component of the model serves to reduce the error (or “noise”) in our estimation of the predictive effect of HLAII alleles for the peptide counts by accounting for the clustering due to individuals, experiments, and isomers. This approach can therefore be understood as maximizing the signal-to-noise ratio. Model 2 was more focused in that the single HLAII allele predictor variable in the fixed-effect component consisted of only DRB1*15:01 and DQB1*06:02. Model 2 was also simpler in that we only accounted for two random effect variables: for individuals (in this case two and six levels for Peyron et al. (2018) and Diego et al. (2020), respectively) and experiments (Peyron et al., 2018; Jankowski et al., 2019; Diego et al., 2020). Thus, Model 2 is a direct head-to-head comparison of the two main HLAII alleles of interest.

Using data from HAPs, we performed a tetrachoric correlation analysis of dichotomous HA status (affected versus unaffected) (N = 71) and FEI status (affected (i.e., FEI+) versus unaffected (i.e., FEI−)) (N = 64) each time with DRB1*15:01 (presence versus absence) in the Psych package in R. Tetrachoric correlation analysis assumes that the dichotomous variables are each underlain by a normally distributed, latent variable, termed the “liability”, along which there is a threshold, where individuals with liabilities greater than the threshold are affected. The 95% confidence intervals (CIs) and *p*-values were obtained by bootstrap on 1,000 bootstrap samples.

To address allele-specific association with FVIII immunodominant epitopes reported in the literature, we performed a Fisher’s Exact test of a 2×2 contingency table in which the exposure row variable is a dichotomous DRB1*15:01 or DQB1*06:02 status (that is, where an amino acid residue is derived from a peptide bound to the HLAII allele or, more accurately, to the HLAII allotype containing the β-chain encoded by the allele), and the outcome column variable is a dichotomous epitope status scored 1 if a particular tFVIII-dP sequence corresponds to a reported epitope, and 0 otherwise. The immunodominant epitopes considered were found to correspond to sequences from the A2, A3, C1, and C2 domains (Hu et al., 2004; Reding et al., 2003; Reding et al., 2004; Jones et al., 2005; James et al., 2007; Ettinger et al., 2010; Ettinger et al., 2016). Thus, with respect to the dichotomous epitope variable, amino acid residues falling within the published immunodominant epitope sequences were scored 1, and those falling outside those sequences were scored 0.

## Results and discussion

**FIGURE 1:**
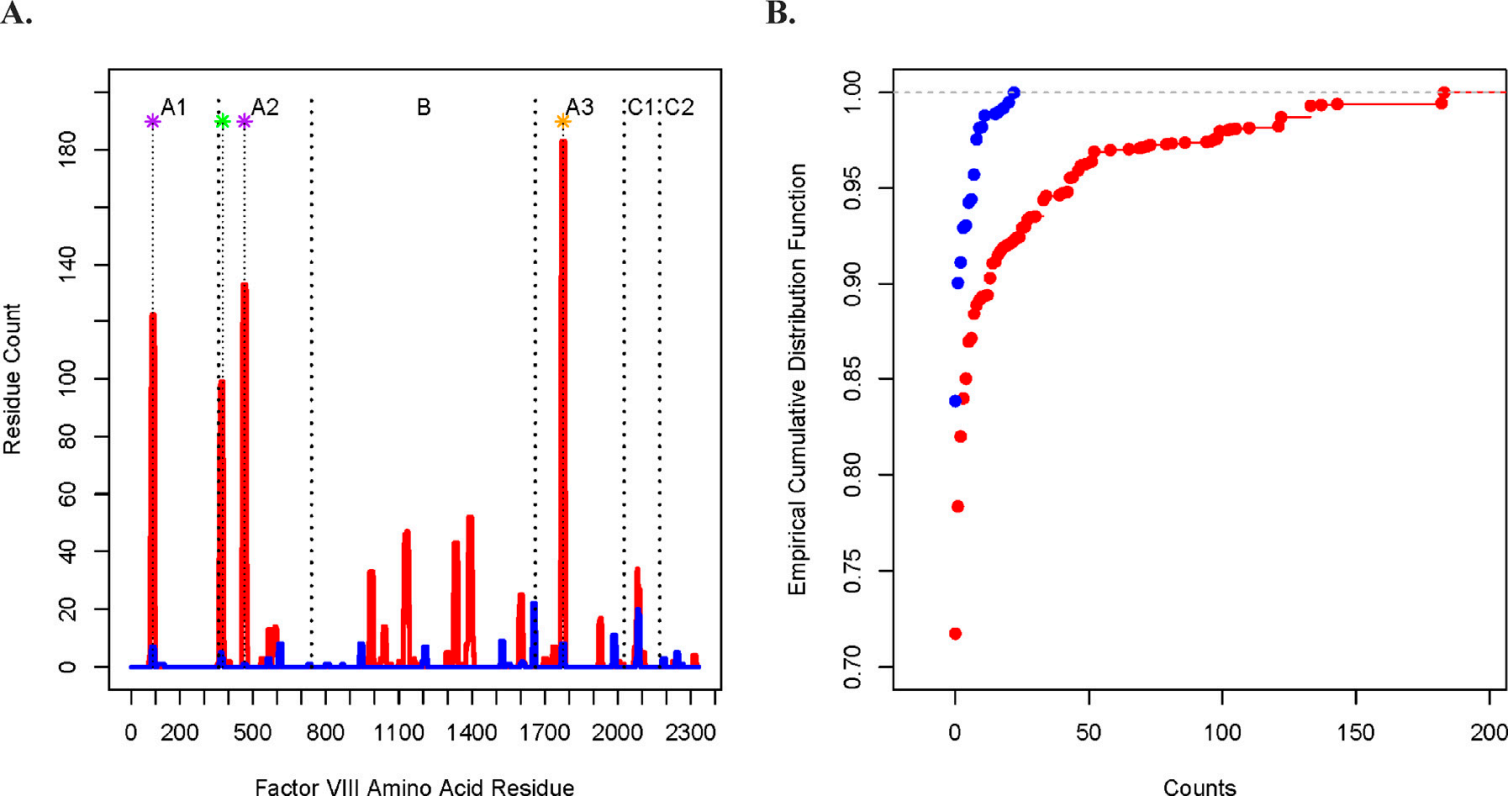
Comparison of the DQ- and DR-derived peptide counts. **(A)** Peptide counts of the DQB1 (blue) and DRB1 (red) fractions at each amino acid position along the FVIII protein, where the A1, A2, B, A3, C1, and C2 domains are indicated at the top of the figure and where the vertical dashed lines mark contiguous domains. The asterisks at the top indicate the midpoint of ranges from the N-termini to the C-termini of the tFVIII-derived IPSs (relative to the amino acid sequence of the FL-rFVIII parent protein) reported in other studies of potentially immunogenic peptides as well as the corresponding locations from the current study. The purple asterisks indicate peptides that were reported in van Haren et al. with residue midpoints—after rounding to the nearest integer—of 89 and 466 (van Haren et al., 2011; van Haren et al., 2013). The green asterisk located at residue 376 represents a peptide in van Haren et al. (2011), van Haren et al. (2013), Sorvillo et al. (2016), and Hu et al. (2004). The study by Hu et al. (2004) is of interest because they demonstrated in CD4 T-cell stimulation assays that their reported peptide, which spanned residues 371 to 400, consistently had the highest immunogenicity index in NDs as well as in HAPs both those with (FEI+) and without (FEI−) FEIs. The orange asterisk located at midpoint residue 1775 represents a peptide reported in van Haren et al. (2011), van Haren et al. (2013), Sorvillo et al. (2016), and Reding et al. (2004).

In Figure 1A, we plotted the peptide count (per amino acid residue along the tFVIII primary sequence). In Figure 1B, we compared the empirical cumulative distribution functions (eCDFs) of the DQB1 and DRB1 counts. The two are significantly different by a two-sided Kolmogorov–Smirnov test (p < 0.0001) of the difference of eCDFs.

**FIGURE 2:**
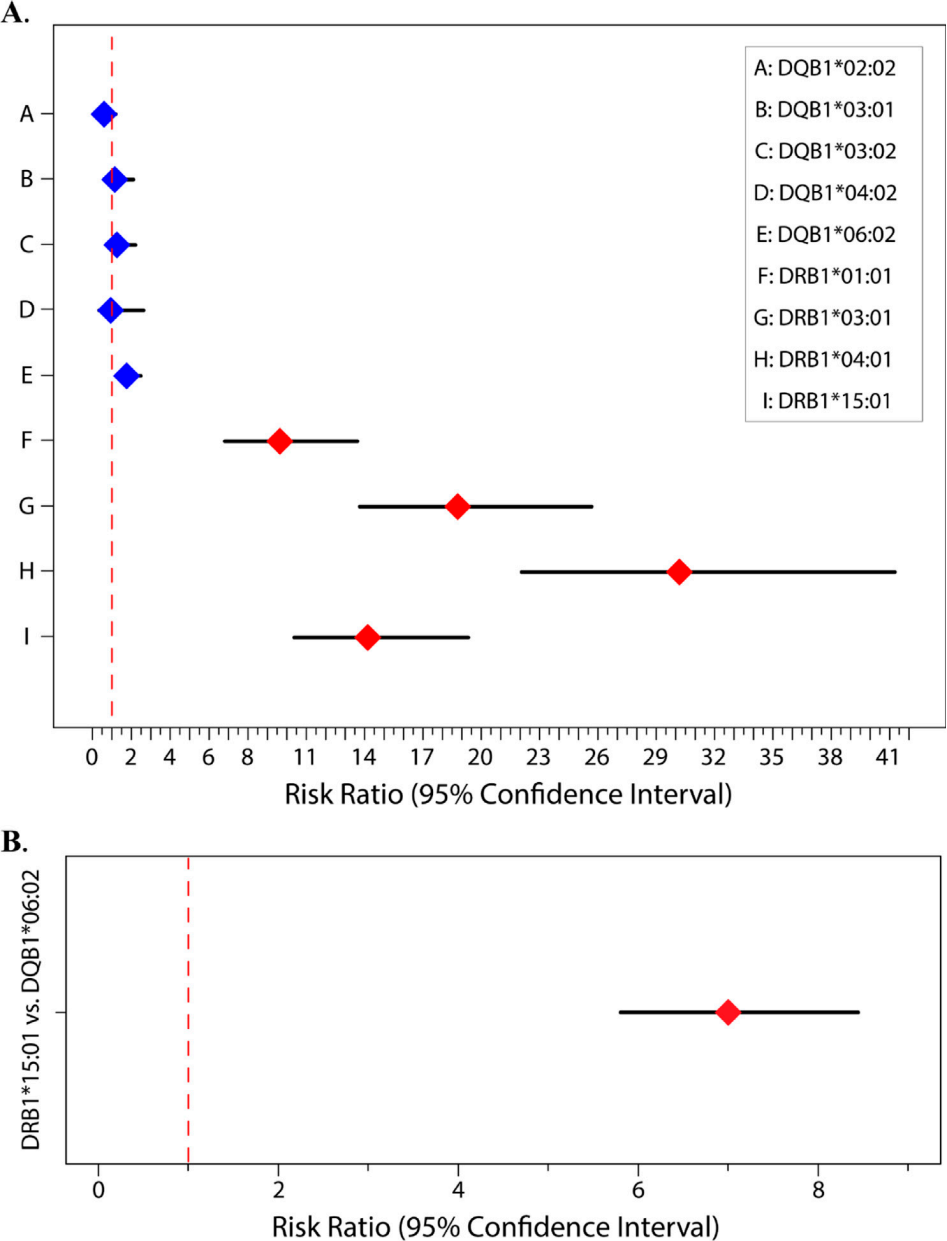
Risk ratios for **(A)** Model 1. *DRB1* alleles (red) and *DQB1* alleles (blue) with DQB1*02:01 being the baseline (i.e., reference) allele; **(B)** Model 2. Head-to-head comparison of DRB1*15:01 (red) to DQB1*06:02 with DQB1*06:02 being the baseline/reference.

The Model 1 results are reported in Figure 2A and Table 1A, where relative to the baseline reference allele provided by DQB1*02:01, both DRB1*15:01 and DQB1*06:02 are at significantly increased risk of contributing to the overall peptide count. Results are reported as risk ratios (RRs) and their associated 95% CI lower and upper bounds (95% CI LB and 95% CI UB). For DRB1*15:01 and DQB1*06:02 respectively, we found an RR (95% CI LB, 95% CI UB) of 14.16 (10.38, 19.33) and 1.76 (1.24, 2.50). Because they are compared to the same baseline, the two RRs may also be directly compared, thus showing that DRB1*15:01 contributes significantly more to the overall peptide count than DQB1*06:02. Under Model 2 (the head-to-head comparison), the RR for the DRB1*15:01 allele against the baseline DQB1*06:02 allele is 7.00 (5.80, 8.44) as reported in Figure 2B and Table 1B.

**TABLE 1:** Risk ratios and their 95% confidence intervals (CIs) under (**A**) Models 1 and (**B**) 2.

HLAII (Gene*Allele)	Risk ratio	95% CI (LB)	95% CI (UB)	p-value
A. Model 1				
DQB1*02:02	0.60	0.30	1.20	1.50 × 10^−1^
DQB1*03:01	1.15	0.63	2.11	6.50 × 10^−1^
DQB1*03:02	1.26	0.71	2.21	4.29 × 10^−1^
DQB1*04:02	0.94	0.34	2.63	9.09 × 10^−1^
DQB1*06:02	1.76	1.24	2.49	1.56 × 10^−3^
DRB1*01:01	9.64	6.81	13.63	1.59 × 10^−37^
DRB1*03:01	18.79	13.75	25.68	1.04 × 10^−75^
DRB1*04:01	30.19	22.08	41.27	3.70 × 10^−101^
DRB1*15:01	14.16	10.38	19.33	1.21 × 10^−62^
B. Model 2				
DRB1*15:01	7.00	5.80	8.44	4.57 × 10^−92^

**FIGURE 3:**
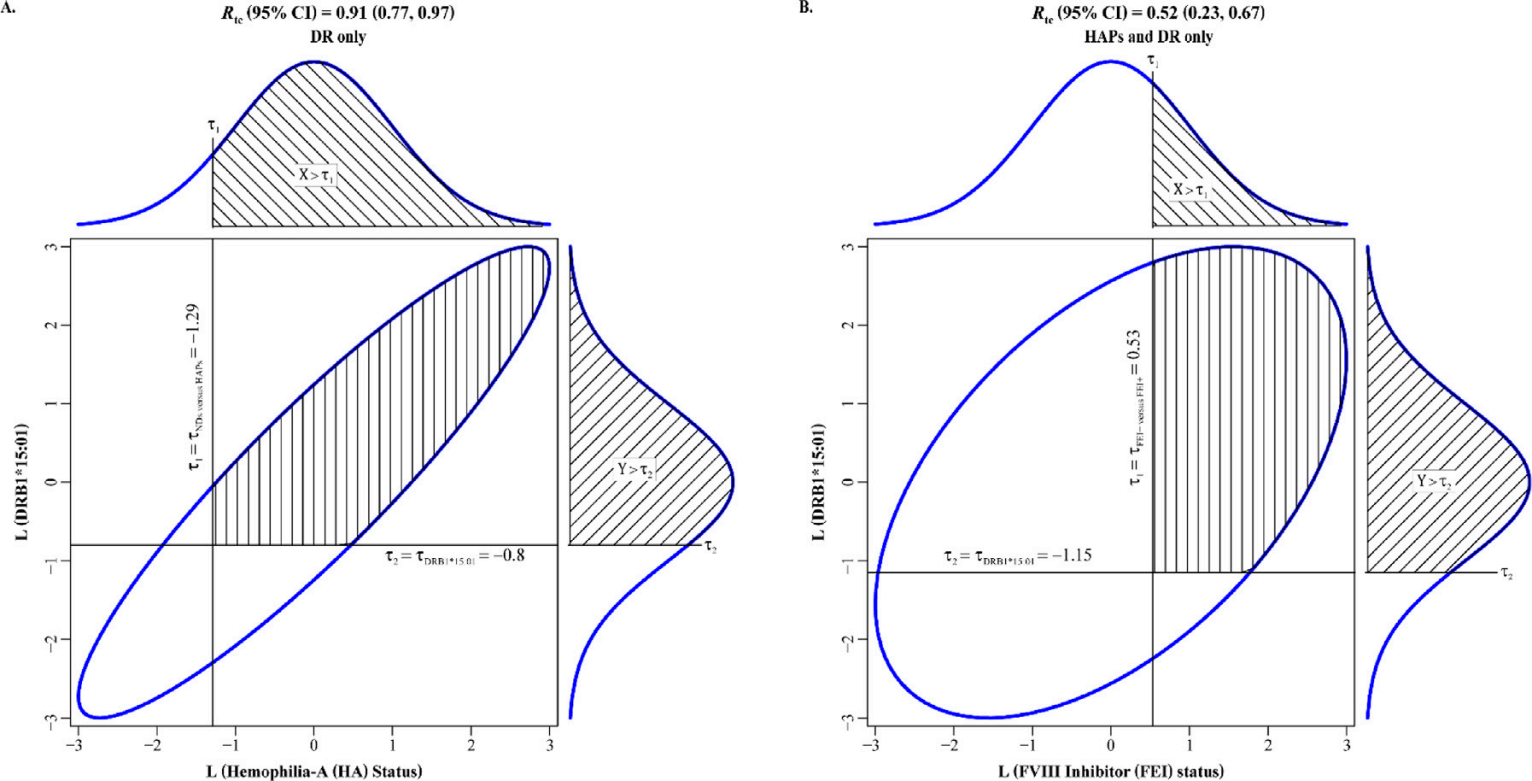
Tetrachoric correlations between DRB1*15:01 (presence versus absence) with **(A)** hemophilia-A (HA) status (affected versus unaffected) and **(B)** FVIII inhibitor (FEI) status (yes versus no). Liabilities to HA status and FEI status are on the horizontal axes in **(A)** and **(B)**, espectively, whereas liability to DRB1*15:01 is on the vertical axis in **(A)** and **(B)**. The ellipses are the 95% confidence intervals (CIs) for the bivariate normal of the two latent, normally distributed liabilities in **(A)** and **(B)**.

We now report the tetrachoric correlation analysis results in terms of the estimate and the LB and UB of its 95% CI, followed by its *p*-value, in parentheses. We found tetrachoric correlations of 0.91 (0.77, 0.97; 3.7 × 10^−4^) for HA status and DRB1*15:01 (Figure 3A) and 0.52 (0.23, 0.67; 2.9 × 10^−5^) for FEI status and DRB1*15:01 (Figure 3B).

Results from the statistical analysis of the differential association of DRB1*15:01 and DQB1*06:02 alleles with known immunodominant epitopes in tFVIIIs are shown in Table 2. We found an odds ratio of 1.35 with a 95% CI of 1.10–1.68 (p = 0.005). The inference is that the DRB1*15:01 allele is significantly more associated with epitopes than the DQB1*06:02 allele.

**TABLE 2:** Differential association of DRB1*15:01 and DQB1*06:02 with epitopes.

Allele	Epitope status	
	1	0
DRB1*15:01	243	1,680
DQB1*06:02	169	1,583

## Conclusion

The following two limitations should first be mentioned: (1) These results are from data obtained from DC-PPPAs deriving from Caucasian subjects (or predominantly Caucasian subjects) and so are not generalizable to all groups of people; and (2) Functional T-cell studies are needed to confirm the causality of the DRB1*15:01 allele.

It appears that the strong immunogenicity of the DRB1*15:01/DQB1*06:02 haplotype generally and the DRB1*15:01 allele specifically is underlain at the molecular and cellular levels by a differentially high (i) affinity of DR15 molecules for tFVIII-dPs and (ii) level of DC (and B cell) presentation of DR15-bound/tFVIII-derived peptide complexes to naïve tFVIII-specific CD4 T cells (and to tFVIII-specific helper T cells). The development of FEIs, and thus FEI risk, is complicated as it involves the humoral component of the adaptive immune system and its interactions with the DCs of the innate immune system, which we have hypothesized is regulated by the individually distinct repertoires of HLAII molecules expressed by HAPs via an immunologically relevant gatekeeper role (Diego et al., 2020). Our findings help further characterize the molecular determinants of FVIII immunogenicity as well as the role of HLAII genetics (alleles, genotypes, haplotypes, and allotypes) in the pathogenesis of FEIs, and also provide data to guide the development of precision medicine approaches to improve the management of HAPs. Further investigation is needed to determine the role of these HLAII alleles, genotypes, and haplotypes, as well as HLAII allotypes and isomers, in other diseases, such as the development of multiple sclerosis, narcolepsy, and drug-induced-hypersensitivity with liver injury.

## Data Availability

The raw data supporting the conclusions of this article will be made available by the authors, without undue reservation.

## Glossary

CI: Confidence interval
DCs: Dendritic cells
eCDFs: Empirical cumulative distribution functions
F: Factor
FVIII: Factor VIII
FVIII:C: FVIII coagulant activity
FED Study: FVIII Epitope Determination Study
FEIs: FVIII inhibitors
FEI−: Negative for FEIs
FEI+: Positive for FEIs
*F8*: FVIII gene
FL: Full-length
HA: Hemophilia A
HAPs: HA patients
HLA: Human leukocyte antigen
HLAII: HLA-class-II
IMD: Immune-mediated disease
IP: Immunogenic potential
IPSs: Individual peptide sequences
LD: Linkage disequilibrium
LC: Liquid chromatography
LB: Lower bound
MHC: Major histocompatibility complex
MAPPs: MHC-associated peptide proteomics
MS: Mass spectrometry
MS/MS: Tandem MS
−: Negative
NDs: Normal donors
ns: Nonsynonymous
ORs: Odds ratios
PBMCs: Peripheral blood mononuclear cells
pd: Plasma-derived
PPPAs: Protein processing and presentation assays
QC: Quality control
RRs: Risk ratios
+: Positive
r: Recombinant
SNVs: Single-nucleotide variations
tFVIII: Therapeutic FVIII protein
tFVIII-dPs: tFVIII-derived peptides
UB: Upper bound
